# Effect of a Milk Protein Concentrate Supplement on Muscle Recovery and Oxidative Stress Following Knee and Hip Arthroplasty: A Randomized Control Trial

**DOI:** 10.3390/antiox15060706

**Published:** 2026-06-03

**Authors:** Maria Spanoudaki, Stavros Kalogiannis, Antonios Cheimaras, Dimitrios Georgianos, Stavros Pellios, Kyriaki Petridou, Thomas Apostolou, Constantinos Giaginis, Sousana Konstantinos Papadopoulou

**Affiliations:** 1 Department of Nutritional Sciences and Dietetics, School of Health Sciences, International Hellenic University, 570 01 Thessaloniki, Greece; kalogian@ihu.gr (S.K.);; 2 Second Orthopedic Clinic, 424 Military Hospital of Thessaloniki, New Efkarpia Ring Road, 564 29 Pavlos Melas, Greece; dimgeo.ortho@gmail.com; 3 First Orthopedic Clinic, 424 Military Hospital of Thessaloniki, New Efkarpia Ring Road, 564 29 Pavlos Melas, Greece; 4 Department of Nutritional Sciences and Dietetics, School of Health Sciences, Hellenic Mediterranean University, 723 00 Sitia, Crete, Greece; kikipetridou2001@gmail.com; 5 Department of Physiotherapy, School of Health Sciences, International Hellenic University, 570 01 Thessaloniki, Greece; apostolouthomas@hotmail.com; 6 Department of Food Science and Nutrition, School of Environment, University of Aegean, 814 00 Myrina, Limnos, Greece

**Keywords:** milk protein concentrate, arthroplasty, osteoarthritis, skeletal muscle mass, CRP, 8-isoprostanes

## Abstract

Background: Postoperative rehabilitation following Total Knee or Hip arthroplasty (TKA, THA respectively) for end-stage osteoarthritis is frequently characterized by oxidative stress and chronic-inflammation-induced muscle atrophy. This study investigated the efficacy of a milk protein concentrate supplement (MCPS) on oxidative stress, inflammation markers, and functional regains in patients undergoing TKA or THA. Methods: 88 participants (aged 55–80 years) were allocated to either an Intervention Group (IG, n = 44), receiving the MPCS, or a Control Group (CG, n = 44), following conventional nutrition for 15 weeks. Appendicular skeletal muscle mass (ASMM) was measured using bioelectrical impedance analysis and functionality through handgrip strength, gait speed, and static balance. 8-Isoprostane levels were quantified in plasma samples using the Enzyme-Linked Immunosorbent Assay(ELISA) method. C Reactive Protein (CRP) levels in serum specimens were measured. Data analysis was conducted, with adjustments made for age, gender, and comorbidities. Results: The IG demonstrated a significant increase in ASMM (Adj. mean change, Δ = +2.34 kg, 95% CI: 1.99 to 2.69, *p* < 0.001) and ASMM Index (Δ = +0.82 kg/m^2^, 95% CI: 0.64 to 1.00, *p* < 0.001) compared to the CG. Functional measurements also showed significant improvements in the IG, including Handgrip Strength (Δ = +4.40 kg, *p* < 0.001), Gait Speed (Δ = +0.23 m/s, *p* < 0.001), and the 2-Minute Walk Test (Δ = +12.02 m, *p* = 0.026). Regarding biochemical markers, the IG showed a significant reduction in plasma F2-isoprostane levels (Δ = −29.19, *p* < 0.001), CRP levels (Δ = −0.69 mg/L, *p* = 0.004), and PTH levels (Δ = −27.41 pg/mL, *p* < 0.001). A negative association between lipid peroxidation (8-isoprostanes) and ASMM was confirmed. Conclusions: Structural nutritional intervention can effectively mitigate catabolic stress triggered by surgical treatment. Implementing such strategies into orthopedic care offers a practical approach to treat challenges often associated with postoperative muscle loss.

## 1. Introduction

The clinical treatment of end-stage osteoarthritis (OA), still remains one of the largest challenges in orthopedic practice, with chronic pain and functional limitation impacting patients as early as the sixth decade of life and often leading to extensive joint replacement such as total knee or hip arthroplasty [1]. Though these procedures are highly effective for repairing joint damage and restoring function, the early postoperative period presents multiple acute physiological stressors which may negatively affect recovery in active adults. Recent evidence shows that OA is a low-grade systemic inflammatory disease that goes beyond a mechanical “wear-and-tear” hypothesis, and it can be markedly exacerbated by ischemia-reperfusion injury as well as surgical trauma. Thus, the early application of qualitive postoperative recovery is imposed by the dynamic between systemic inflammatory features (e.g., C-reactive protein (CRP)) and oxidative stress markers such as F_2_-isoprostanes [2,3].

During the surgical window period, a major biological barrier is the upregulation of reactive oxygen species (ROS). The F_2_-isoprostanes (F_2_-IsoPs) are derived from the non-enzymatic peroxidation of membrane lipids, and provide the most reliable “gold standard” assessment of this oxidative injury in vivo [3]. The oxidative stress is closely associated with the inflammatory response. High levels of interleukin-6 (IL-6) induce CRP hepatic synthesis, a cascade that can severely hinder tissue healing, prolong rehabilitation, and increase pain postoperatively [2]. Additionally, the stress from surgical operation and early postoperative immobilization stimulates the Ubiquitin-Proteasome pathway. Ubiquitin ligases label myofibrillar proteins for degradation, causing loss of muscle mass and power to occur at an increased rate even in the relatively non-geriatric population while glutathione synthesis exhibits a pivotal protective role against this downregulation [4]. To treat these catabolic pathways, targeted nutrition has emerged as a pillar in orthopedic care. While the literature traditionally focuses on the role of alpha-linolenic acid in inflammatory modulation, the structural integrity of the musculoskeletal system during recovery is incredibly dependent on mineral homeostasis and an optimized protein matrix. The efficacy of these nutrients depends on their synergistic ability to mitigate “muscle-bone crosstalk” and support neuromuscular function.

In this context, the use of a specialized milk protein concentrate (Protifar, Nutricia) provides a biphasic release of amino acids, whey for rapid glutathione (GSH) synthesis, and casein for long-lasting anabolic support [5], while maintaining a favorable electrolyte balance. The milk-derived proteins are also crucial in managing sarcopenia via mTOR activation signaling pathway [6]. However, ROS-induced muscle atrophy remains a barrier [7]. High-protein diets are essential to counteract fat-free mass loss [8], as amino acids are essential for skeletal muscle maintenance and function [9]. The ingestion of casein in a milk matrix modulates digestion kinetics and absorption, offering sustained support [10,11].

Recent evidence suggests that an optimized dietary Ca/P ratio is a major factor in determining bone health [12]. Therefore, providing a Ca/P ratio close to 1.9:1 ensures the ionic availability necessary for excitation-contraction coupling and osseointegration. In this way, secondary hyperparathyroidism is ensured and consequent bone resorption caused by a high phosphorus diet is avoided [13,14]. This is also enhanced by muscle–bone crosstalk [15]. Moreover, electrolyte balance, such as Sodium (Na) and Potassium (K), is also important in maintaining the resting membrane potential in myocytes and in avoiding postoperative fluid retention that may impair early mobilization. The low electrolyte content in the chosen matrix ensures compatibility with clinical guidelines for patients requiring electrolyte balance and fluid management in the postoperative period, supporting the Na^+^/K^+^-ATPase pump and avoiding muscle fatigue [16]. This is important in order to prevent disuse-induced muscle wasting [17] and electrolyte imbalance [18,19].

The primary aim of this randomized control trial was to investigate the efficacy of a targeted nutritional intervention on Appendicular Skeletal Muscle Mass (ASMM), in patients undergoing primary knee or hip arthroplasty due to end-stage osteoarthritis. Furthermore, the effects on oxidative and inflammation markers were determined in order to elucidate the underlying cellular, physiological, and biochemical mechanisms associated with improvements in functional recovery.

## 2. Materials and Methods

### 2.1. Study Design

Sample size was determined by conducting a priori analysis, using G Power software (version 3.1, Heinrich Heine University, Düsseldorf, Germany; developed by Faul et al.).

This two-armed, single-blind randomized controlled trial enrolled 88 participants (aged 55–80 years), who underwent elective total knee or hip arthroplasty due to end-stage osteoarthritis. Participants were randomly allocated to either the Intervention Group (IG) or Control Group (CG) using a computer-generated randomization list in Microsoft Excel. Especially, the random allocation sequence was created by using Microsoft Excel function as follows: =RAND(), producing a uniform distribution of decimal numbers. Each individual was allocated by getting a random decimal value. The dataset was sorted by these values to create a distribution rate 1:1 (44 participants per group). Participants were randomly allocated to either the Intervention Group (IG) or the Control Group (CG) in a 1:1 ratio (44 participants per group). Randomization was performed using a computer-generated sequence in Microsoft Excel (via the =RAND() function), which produced a uniform distribution of decimal values used to sort and assign individuals.

Also, participants were enrolled in sequential blocks aligned with the hospital’s twice-weekly surgical schedule. This approach was chosen to ensure strict adherence to the nutritional protocol and to prevent cross-contamination between groups in a shared clinical environment. The CG and IG were recruited during sequential, non-overlapping periods to prevent contamination bias, which can occur when participants in the same clinical ward communicate and compare nutritional protocols. To ensure consistency and eliminate confounding factors, both recruitment phases were conducted within the same clinical setting, involving the same surgical and nursing teams. Furthermore, all patients followed the identical standardized clinical pathway and postoperative rehabilitation protocol, ensuring that the transition between time periods did not introduce variations in the quality of care or treatment delivery.

While participants were aware of their group allocation due to the nature of the nutritional intervention, a rigorous blinding protocol was enforced for the rest of the research team. Specifically, the clinical assessors (physiotherapists and physicians), the principal investigator, and the statisticians remained strictly blinded to group allocation throughout the entire study and data analysis phase. Although a placebo-controlled design was not feasible given the unique nutritional and sensory profile of the specific formula, we took extensive measures to ensure objective results. To this end, both groups followed an identical, supervised physical therapy protocol, ensuring that rehabilitation adherence remained consistent across the study population and did not act as a confounding factor.

The research personnel determined the presence of clinical depression or dementia and comorbidities during the initial screening, based on a detailed review of the patients’ medical records.

We excluded patients with malabsorption disorders; the presence of a pacemaker and/or Implantable Cardioverter Defibrillator; known allergies to soy or milk proteins; significant renal, hepatic, or cardiac failure; active malignancy; and neuro-musculoskeletal or severe psychiatric disorders (e.g., clinical depression or dementia) that could limit the participants’ ability to comply with the dietary protocol or provide informed consent. The study protocol was approved by the participated Hospital Ethics Committee (45/24 July/6th session/2023), and all subjects provided written informed consent.

### 2.2. Physical Therapy

All participants underwent a standardized physical therapy program focused on isometric exercises and progressive resistance training. The rehabilitation process was divided into two phases. Phase I (Inpatient) commenced on the first postoperative day and focused on early mobilization, respiratory exercises, and basic isometric contractions to prevent complications. Upon hospital discharge, all participants entered Phase II (outpatient), consisting of three supervised sessions a week (45 min each) for 5–6 months. This second phase followed a progressive resistance training model combined with balance and gait stabilization exercises. Compliance was strictly monitored through hospital attendance logs and therapist progress reports. Each group received standard physical therapy instructions and supervision to ensure that the nutritional intervention was the only factor affecting muscle recovery outcomes.

### 2.3. The Nutritional Protocol

The study design ensured that the groups remained independent; the CG was unaware of the supplementation protocol, effectively eliminating contamination bias that usually occurs when patients compare treatments.

The supplementation protocol was implemented during the early postoperative period and was maintained for 15 weeks, while the CG received no supplement. The absence of a placebo also was due to ethical considerations regarding informed consent and the technical difficulty of providing a sensory-matched non-protein substitute in a clinical setting. To ensure group independence and eliminate contamination bias—common when patients in the same ward compare treatments—the CG and IG were recruited during sequential, non-overlapping periods. Participant compliance was a main priority. In the IG, adherence was ensured through 3 days of food intake diaries and cross-verified by collecting empty supplement canisters at follow-up. The supplement’s neutral flavor significantly aided adherence, as it was easily incorporated into water, juices, or meals without compromising palatability.

Overall energy intake was standardized for both groups, with a total protein target of 1.3 g/kg/day, according to ESPEN guidelines for postoperative recovery [20]. For the IG, 30 g of this requirement was provided via the milk protein concentrate supplement.

The CG met the same protein targets exclusively through regular dietary sources, without using a supplement or a placebo substitute. CG’s compliance was also ensured via 3 days of food intake diaries.

Energy requirements were calculated individually to avoid overfeeding and metabolic instability, implementing a rate of energy intake in the range of 25–30 kcal/kgr [20]. Both groups followed a Mediterranean diet pattern. Τhus, all participants received personalized dietary advice and a structured meal plan. Adherence to the diet and the prescribed protein intake (1.3 g/kg/day) was monitored at each follow-up visit through the evaluation of 3 days of food diaries, which were reviewed by a dietitian to ensure consistency with the provided dietary guidelines at each follow-up visit to confirm that both groups adhered to the Mediterranean-style diet and reached the isoenergetic goals. Statistical analysis of the dietary records confirmed that there were no significant differences in total daily energy intake (kcal) or total daily protein intake (g/kg/day) between the IG and the CG throughout the 15-week period (*p* > 0.05). Thus, both groups remained strictly isoenergetic and protein-matched, and the CG successfully met their nutritional targets exclusively through regular food sources without protein intake disparities.

This qualitative monitoring confirmed that participants in both groups maintained stable dietary patterns, ensuring that the study’s primary outcomes were not influenced by significant deviations in total energy intake or dietary composition.

To support the recovery process and address the increased metabolic demands following surgery, we implemented a specific nutritional intervention. The goal was not only to meet protein targets but to strategically time the intake to maximize muscle protein synthesis and support bone health.

Participants in the IG received a daily supplementation of 30 g of milk protein concentrate (Protifar, Nutricia). This was administered using 12 precision scoops (2.5 g each) and was divided into two strategic doses to optimize its biological impact, as follows:

Morning Administration: 15 g (6 scoops) were taken with breakfast to decisively reverse the muscle protein breakdown (catabolism) that occurs during the overnight fast.

Post-Rehabilitation Administration: 15 g (6 scoops) of the milk protein supplement was administered within 30 min following either the supervised sessions or the home-based resistance exercises to consistently exploit the post-exercise anabolic window. This timing was chosen to exploit the “anabolic window,” providing the necessary substrates for muscle repair when the body’s sensitivity to amino acids is at its peak.

The choice of Protifar was based on its protein profile, providing 27.5 g of pure protein per daily dose. By utilizing a milk protein concentrate, we provided an almost 80:20 ratio of casein to whey protein. This combination offers a “fast-slow” diphasic action, as follows: the whey fraction (~5.5 g) stimulates an immediate anabolic response, while the casein fraction (~22 g) provides a sustained release of amino acids into the bloodstream, protecting muscle tissue for several hours. Beyond muscle maintenance, the protocol addressed the structural needs of lower-limb arthroplasty recovery. The daily 12-scoop regimen provided 408 mg of calcium with an optimal calcium-to-phosphorus (Ca:P) ratio of 1.9:1. Furthermore, the supplement contains soy lecithin as an emulsifying factor and an omega6/omega3 ratio of 3.3:1, ensuring that the nutritional support was in line with an anti-inflammatory dietary profile. Also, the osmolarity of the supplement was 25 mOsmol/L. No adverse effects were reported.

### 2.4. Laboratory Procedures

Giving priority to patients’ comfort, blood samples were obtained by utilizing aliquots from routine preoperative and follow-up examination screenings. This approach ensured that no additional invasive procedures were required for the study’s measurements.

#### Blood Samples

Venous blood was collected using two separate vacuum tubes to accommodate the specific requirements of each analysis. Samples intended for the measurement of oxidative biomarkers were collected in EDTA-treated tubes and were immediately centrifuged at 3000 rpm (1500× *g*) for 15 min at 4 °C. The resulting plasma aliquots were flash-frozen and stored at −80 °C until the final analysis of 8-isoprostane.

Simultaneously, blood serum for the biochemical profile (urea, creatinine), C-reactive protein (CRP), and Parathormone (PTH) was collected in clot-activator tubes. These samples were allowed to clot at room temperature for 30 min before being centrifuged at 3000 rpm for 15 min to obtain the serum.

Just after centrifugation, all plasma and serum sub-samples were hermetically covered with Parafilm M^®^ to prevent ex vivo artifactual oxidation. All samples were then stored at −80 °C to ensure the stability of the oxidative biomarkers until the time of measurement.

Quantification of plasma 8-Isoprostanes(8-iso PGF 2a) was conducted by using the Elisa method. Before the quantification of total 8-isoprostane levels, the plasma samples underwent alkaline hydrolysis to release esterified 8-isoprostane from phospholipids, according to the manufacturer’s protocol (Cayman Chemical, Ann Arbor, MI, USA; Item No. 516351I). For the hydrolysis procedure, 200 μL of plasma was incubated with 200 μL of 2 M NaOH at 45 °C for 60 min. Following incubation, the mixture was neutralized by adding solution of 200 μL HCl 2 M and stabilized with 100 mM phosphate buffer. All samples were analyzed in duplicate. The absorbance was measured at 405 nm using a microplate reader.

Butylated hydroxytoluene (BHT) was added to the plasma aliquots at a concentration of 0.005% just after separation, in order to prevent ex vivo formation of 8-isoprostane because of artifactual lipid peroxidation. The plasma sub-samples were then instantly transferred to an ultra-low temperature freezer and stored at −80 °C until analysis.

Serum electrolytes, and calcium and phosphorus as well, were monitored and remained within normal reference ranges for all participants throughout the study period.

### 2.5. Body Composition and Functional Assessment

Body composition was assessed using the Bodystat 1500 MDD bioelectrical impedance analyzer, a dual-frequency device (5 kHz and 50 kHz), (Bodystat Ltd., Douglas, Isle of Man, British Isles). Measurements were strictly standardized to minimize hydration-related bias, as follows: the baseline assessment was performed on the morning of surgery, following a 12 h overnight fast (nil by mouth, including liquids). Τhe final assessment at the 15th week followed the same protocol, including a 4 h fasting and a 24 h abstinence from both alcohol consumption and vigorous physical exercise [21]. Participants were placed in a supine position for 10 min prior to measurement to allow for fluid stabilization, with electrodes placed according to the manufacturer’s instructions. Calibration was conducted daily and confirmed using the 500-ohm test resistor, while data management and analysis were performed via the Body Manager Pro software version 6.0.1.451 for Windows10, ensuring the application of validated algorithms and preventing potential human error during data transfer from the device. Alongside these measurements, we recorded each participant’s weight and height—using a Tanita WB-380 scale and a built-in stadiometer, respectively—with subjects in light attire and no shoes to ensure an accurate BMI calculation. Body Mass Index (BMI) was calculated as weight in kilograms divided by the square of height in meters (kg/m^2^).

Lower-limb performance was evaluated through a battery of validated tests designed to assess different dimensions of functional capacity. Lower limb power was evaluated using the 5-repetition Sit-to-Stand (STS-5) test according to Bohannon’s protocols [22], while mobility and endurance were evaluated through Gait Speed and the 2-Minute Walk Test (2MWT) [23].

Muscle strength was quantified via handgrip strength using a Takei digital handgrip dynamometer (Takei Scientific Instruments Co., Ltd., Tokyo, Japan), following the standardized protocols recommended by the European Working Group on Sarcopenia in Older People [24]. During the assessment, participants were instructed to exert maximum isometric strength, with the highest value from three consecutive attempts being recorded to ensure measurement accuracy and reliability. Static balanced was also assessed by implementing Single Stance Test (SLS) [25].

This multifaceted approach allowed for a robust correlation between physiological muscle gains and real-world functional improvements.

### 2.6. Statistics

Statistical analysis and graphical representations were conducted using R software (version 4.5.2) and the ggplot2 package. Normality was assessed for all continuous variables using the Shapiro–Wilk test and visual inspection of histograms. Descriptive statistics are presented as mean ± standard deviation (SD) for continuous variables and as frequencies/percentages for categorical ones. Differences between groups at baseline were analyzed using independent-sample *t*-tests for continuous variables and Pearson’s Chi-squared test or Fisher’s exact test where appropriate for categorical variables.

Baseline comparisons between the intervention group (IG) and the control group (CG) were conducted using independent-sample *t*-tests.

The effect of the intervention on primary and secondary outcomes was evaluated using Generalized Linear Models (GLM). In each model, the post-intervention value was set as the dependent variable, while the group assignment (IG vs. CG) and the respective baseline value were included as covariates (ANCOVA-like approach). This method was chosen to adjust for potential baseline imbalances and to estimate the net treatment effect. Models were also adjusted for age, gender, and comorbidities (hypertension, dyslipidemia, diabetes, depression, previous arthroplasty, and smoking status).

The assumptions of the GLM models were verified by analyzing the normality of the residuals through Shapiro–Wilk tests and Q-Q plots, as well as by checking for homoscedasticity using residual-vs-fitted plots. Results are reported as unstandardized regression coefficients (B), indicating adjusted mean changes adjusted mean changes (Δ) with their corresponding 95% Confidence Intervals (CIs) and *p*-values. Statistical significance was set at *p* < 0.05.

We also explored the potential biochemical mechanisms behind our findings, through the development of GLM models to investigate the association between the change (Delta) in oxidative stress biomarkers (plasma F2-isoprostanes) and postoperative ASMM. Findings from these predictive models were visualized using Forest Plots to display adjusted mean changes (Δ) and 95% Confidence Intervals (CIs). Stratified scatter plots with linear regression fits were generated to highlight the group-specific relationship between reduction in oxidative stress and inflammation, and muscle preservation. Statistical significance was set at *p* < 0.05.

## 3. Results

### 3.1. Participants Characteristics and Baseline Comparisons

**Figure 1:**
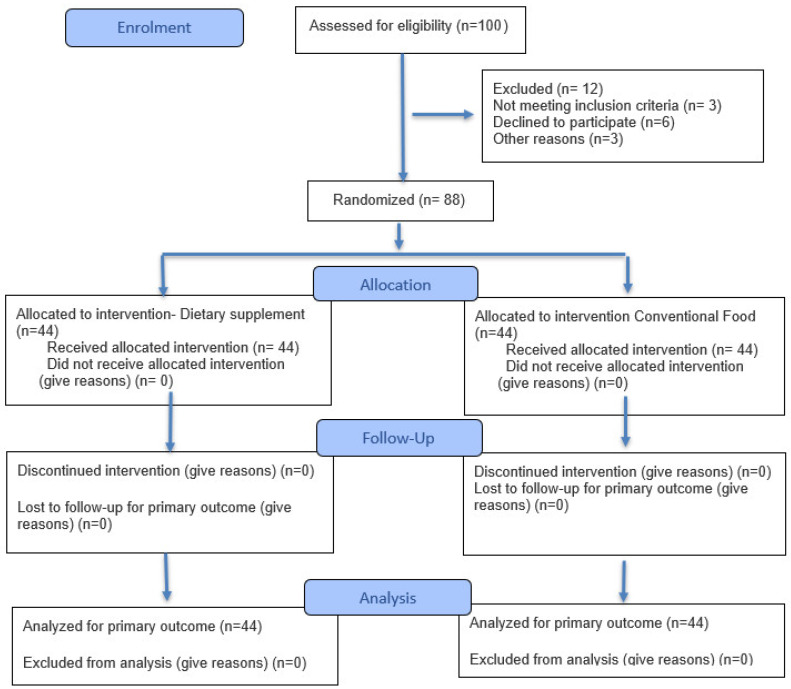
Consort 2025: Flow diagram of the progress through the phases of a randomised trial of two groups (that is, enrolment, intervention allocation, follow-up, and data analysis.

One hundred participants were recruited and screened for eligibility. Twelve of them did not participate, as follows: six refused to participate, three were excluded for personal reasons, and three did not meet all the inclusion criteria (Figure 1). Thus, a total of 88 participants were randomly assigned to two groups at a 1:1 allocation rate. Forty-four of them were assigned to the Interventional Group and 44 to the Control Group, with each group receiving the intended intervention and being analysed for primary outcomes.

Baseline analysis, summarized in Table 1, confirmed that the two groups were well matched at the study entry. No statistically significant differences were observed regarding age, gender distribution (*p* = 0.434), or baseline skeletal muscle mass (*p* = 0.125). Similarly, body composition and primary biochemical oxidative stress and inflammation markers showed no significant differences between groups (*p* > 0.05). Nevertheless, a baseline difference was noted in depression scores (*p* = 0.013), which was subsequently included as a covariate in all adjusted models to ensure the validity of the intervention effects.

#### Dietary Analysis and Macronutrient Distribution

Statistical analysis of the dietary records demonstrated no significant differences in total daily energy intake (kcal) or total daily protein intake (g/kg/day) between the IG and the CG throughout the 15-week period (*p* > 0.05). Thus, both groups remained energy- and protein-matched, and the CG successfully met their nutritional targets exclusively through regular food sources without any energy or protein intake imbalances. In parallel, the IG appropriately adjusted their regular diet to accommodate the intervention, deriving 71.4% of their daily protein from whole food sources and successfully substituting the remaining 28.6% with the provided milk protein concentrate supplement (providing 27.5 g of net protein from 30 g of the supplement powder. The observed statistically significant difference between IG and CG protein intake from conventional food sources is due to the targeted protein replacement from the supplement. The detailed macronutrient composition and the proportion of protein sources for both groups during the study period are summarized in Table 2.

### 3.2. Outcomes Clinical and Functional Outcomes

#### 3.2.1. Body Composition and Muscle-Related Outcomes

Participants in the IG exhibited a substantial increase in Appendicular Skeletal Muscle Mass (ASMM Adj. mean change, Δ = +2.34, SE = 0.18, *p* < 0.001) and the ASMM Index Δ = +0.82, SE = 0.09, *p* < 0.001). Phase Angle, a key marker of cellular health and muscle quality, improved significantly Δ = +0.65°, SE = 0.12, *p* < 0.001), (Table 2).

#### 3.2.2. Biochemical Markers and Oxidative Stress

Our analysis revealed a significant reduction in inflammation and lipid peroxidation markers in the intervention group. As detailed in Table 3, plasma 8 Isoprostanes Δ = −29.19 pg/mL SE = 5.42, *p* < 0.001) showed significant decreases in the IG compared to the CG. CRP levels significantly lowered in the IG following the intervention (1.85 ± 4.10 mg/L vs. 2.45 ± 6.50 mg/L, *p* < 0.004). Correlation analysis confirmed that the reduction in systemic oxidative stress was a key predictor of muscle mass preservation (r = −0.40, *p* < 0.001).

Furthermore, a strong positive correlation was found between PTH and CRP (r = 0.62, *p* < 0.001), indicating a possible linked hormonal-inflammatory response. A General Linear Model (GLM) identified CRP (*p* = 0.003) and plasma 8 Isoprostanes (*p* = 0.008) as the strongest independent predictors of ASMMI at 15 weeks; PTH possibly influences muscle mass indirectly through its association with CRP inflammatory pathways Δ = 0.006, *p* = 0.142).

Moreover, at the 15th week follow-up, the IG showed significantly lowered levels of PTH compared to the CG (69.41 ± 24.10 vs. 94.85 ± 27.90 pg/mL, respectively, *p* < 0.001). Plasma 8 Isoprostanes were also significantly lower in the IG (45.55 ± 28.62 vs. 88.73 ± 53.32 pg/mL, *p* < 0.001). Most importantly, these biochemical improvements indicated that the IG maintained a significantly higher ASMMI compared to the CG (9.85 ± 1.75 vs. 8.35 ± 2.25 kg/m^2^, *p* < 0.001).

#### 3.2.3. Physical Performance and Clinical Symptoms Assessment

Functional capacity improved markedly across most of the assessments. Significant gains were recorded in Handgrip Strength Δ = +4.40, *p* < 0.001) and 4-metre Gait Speed Δ = +0.23, *p* < 0.001). Postural balance, measured by the Single Leg Stance (SLS), improved for the right (Δ = +5.13, *p* < 0.001) and left foot (Δ = +4.85, *p* < 0.001). Additionally, lower-limb power, assessed through the 5 repetitions of Sit to Stand Test, showed significant enhancement (Δ = −3.93, *p* < 0.001), while endurance increased as evidenced by the 2-Minute Walk Test (Δ = +12.02, *p* <0.05).

**Figure 2:**
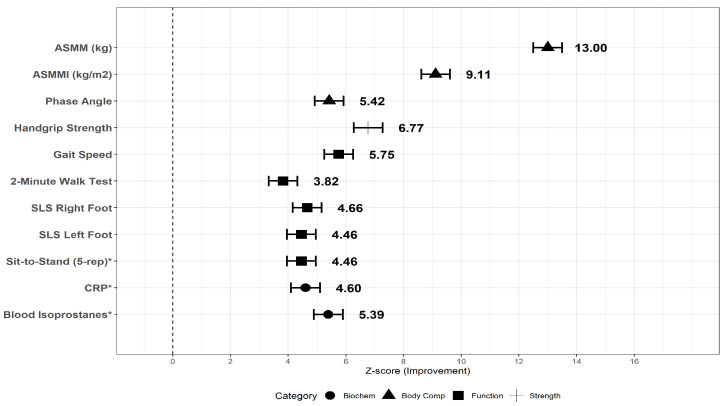
Forest plot of standardized Z-scores reflecting clinical improvement following the 15-week intervention. Estimates were derived from Generalized Linear Models (GLM) adjusted for baseline values, age, smoking, and comorbidities. All parameters are expressed as standardized Z-scores of improvement. Statistical significance (*p* < 0.05) is indicated by the fact that all 95% Confidence Intervals (error bars) do not cross the null effect line (0). Points to the right of the zero line indicate a positive clinical response to the intervention. * Indicates visually the statistical significance; ASMM: Appendicular Skeletal Muscle Mass; ASMMI: Appendicular Skeletal Muscle Mass Index; CRP: C-Reactive Protein; SLS: Single-Leg Stance; Blood Isoprostanes: Plasma 8 Isoprostanes.

To facilitate comparison across outcomes with different measurement units, standardized effect sizes are presented in Figure 2. All parameters showed a significant positive clinical improvement in response to the intervention (Z-scores > 0), with the most pronounced improvements observed in ASMM and ASMMI (Figure 2).

**Figure 3:**
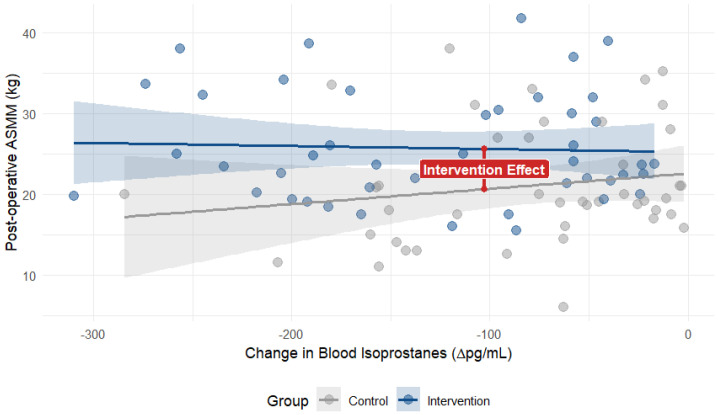
Impact of oxidative stress reduction on postoperative ASMM. Stratified regression analysis showing the association between plasma 8-Isoprostane (referred blood isoprostanes in the plot), reduction (Δpg/mL)) and postoperative skeletal muscle mass. Intervention Group (blue) demonstrates higher ASMM retention across the spectrum of oxidative stress changes compared to the Control Group (gray). The intervention effect was estimated using a multivariate General Linear Model (GLM) adjusted for baseline plasma isoprostane levels, age, gender, and comorbidities, confirming that the observed differences are independent of baseline status. Shaded areas represent 95% confidence intervals. ASMM: Appendicular Skeletal Muscle Mass; IG = Intervention Group; CG = Control Group.

According to the multivariate analysis (Table 2), the IG demonstrated a significant improvement in all primary and secondary outcomes compared to the control group. Specifically, the IG showed a substantial preservation of skeletal mass Δ = 2.34, *p* < 0.001) alongside a significant reduction in systemic oxidative stress markers, with blood plasma 8-Isoprostanes levels decreasing by 29.19 units relative to the CG (*p* < 0.001). In order to assess the relationship between these biological changes, a stratified scatter plot analysis was performed (Figure 3), The analysis indicated that patients in the IG showed both reduced oxidative stress and muscle mass retention compared to the CG.

## 4. Discussion

The present study demonstrates that a targeted intervention significantly enhances appendicular muscle mass and muscle performance, reducing systemic oxidative stress and inflammation. It is worth mentioning that the observed improvements in the intervention group were achieved within the context of an isonitrogenous diet and a standardized physical therapy program, which were followed by both groups. As total protein intake (1.3 g/kg BW/day) and rehabilitation load were balanced between the IG and CG, our research indicates that the greater preservation of muscle mass is potentially due to the particular nutritional composition of the supplement and its effect on the PTH-CRP axis rather than quantitative differences in protein or exercise volume.

Studies have suggested that maintaining appendicular lean mass is a key factor for joint stability, as periarticular muscles act as stabilizers absorbing mechanical loads [26,27]. The significant increase in ASMM observed in our cohort may be attributed to the “muscle-joint axis” theory, according to which improved muscle quantity and quality lead to a substantial reduction in the clinical symptomatic burden [28]—a relationship consistent with the findings of the aforementioned studies. 

A fundamental driver underlying this recovery may be associated with the observed decrease in plasma 8-Isoprostanes levels, which are an index of systemic lipid peroxidation [3,29,30]. Isoprostanes are formed non-enzymatically when reactive oxygen species attack polyunsaturated fatty acids in muscle cell membranes. Excessive lipid peroxidation stimulates the Ubiquitin-Proteasome System (UPS) and calpain activation, leading directly to the degradation of myofibrillar proteins like myosin and actin as well as to chondrocyte degradation [31,32]. In addition, oxidative stress markers, such as isoprostanes, are closely linked to the activation of atrogenes (atrophy-related genes), which orchestrate the breakdown of contractile proteins. Our results suggested that the intervention regulated oxidative impact, mitigating atrogene-mediated myofibrillar degradation and creating an appropriate redox microenvironment for the observed regain in skeletal muscle mass [32]. This biochemical protection was further evidenced by the improvement in Phase Angle, a bioelectrical marker of cellular health and integrity [33]. Additionally, the reduction in CRP levels highlights the anti-inflammatory outcome of the intervention, interrupting the “vicious cycle” between inflammation and oxidative stress [34].

Improved functional outcomes such as gain in strength may be attributed to a faster body transition from a catabolic to an anabolic state, leading to muscles recovering and regaining strength earlier. The gain in strength, particularly in Type II muscle fibers which are affected by aging [35], was probably improved by the protein profile and the specific nutritional composition of the supplement, including its optimal calcium/phosphorus ratio, which enhances excitation-contraction coupling and also supports the mineral environment necessary for both muscle contractility and bone remodeling [18]. Moreover, energy requirements were strategically scheduled individually, according to the latest ESPEN guidelines for surgical and older adult patients [20,36], promoting early recovery.

The metabolic efficiency of this recovery was also influenced by the protein source. While the CG consumed high-quality animal protein from conventional foods, they likely faced “anabolic resistance”, a common barrier in the elderly that suppresses the muscle anabolic response to conventional meals [37]. In contrast, the IG benefited from the continuous amino acid supply by the milk protein concentrate formula [38], which is more effective at providing muscles mainly with glutamine and leucine for protein synthesis than slower-digesting whole food proteins [39,40]. Specifically, the whey fraction provides a rapid leucine spike that acts as a potent trigger for the mechanistic target of rapamycin (mTOR) pathway, initiating muscle protein synthesis (MPS) [5,39]. Simultaneously, the sustained release of amino acids from the casein fraction helps suppress the ubiquitin–proteasome pathway, which is the primary driver of muscle protein breakdown (MPB) during the catabolic postoperative phase [17,40]. This dual action is more effective at providing muscles mainly with glutamine and leucin. Thus, the findings of the present study supported our initial hypothesis that high quality-protein nutritional intervention could efficiently mitigate the ‘anabolic resistance’ often observed in older adults [37,41]. By providing a continuous amino acids supply the intervention successfully overcame the metabolic barriers that typically suppress muscle protein synthesis in the older adults after joint replacement [39,40].

This specialized nutritional support, maintained over the 15-week period, complemented a rehabilitation program of three (3) supervised sessions per week, and daily home-based exercise provided an anabolic stimulus that whole-food diets often fail to achieve in postoperative older adults and elderly cohorts [41,42]. This process is further facilitated by the muscle–bone crosstalk, where myokines from active appendicular skeletal muscles support structural stability and promote bone remodeling [15]. Additionally, the low sodium and potassium profile of the milk protein concentrate supplement supports the stability of the Na^+^/K^+^-ATPase pump, while the maintenance of the sarcolemmal membrane is essential for muscle fiber excitability, optimizing the excitation-contraction coupling and reducing muscle fatigue during the early postoperative phase [16].

Clinically, the increase in gait speed and Single Leg Stance (SLS) scores show the functional regain of the gluteus medius and psoas major [43]. By restoring periarticular strength and optimizing the mineral environment for osseointegration [44], the metabolic cost of the movement decreased.

Furthermore, the multivariate GLM analysis for the 2-Minute Walk Test (2MWT) confirmed that the intervention maintained a significant independent effect (Δ = 12.02, *p* = 0.026), suggesting better metabolic economy of walking, and by regaining periarticular strength, the intervention effectively raised the pain threshold by desensitizing peripheral pain receptors [28].

The significant preservation of ASMMI in the IG (9.85 ±1.75 vs. 8.35 ± 2.25 kg/m^2^, *p* < 0.001) could be attributed to the successful modulation of the hormonal and inflammatory environment. Specifically, the reduction in Parathyroid Hormone (PTH) levels for the IG (69.41 ± 24.10 vs. 94.85 ± 27.90 pg/mL, *p* < 0.001) suggests a stabilized mineral homeostasis. Τhis is particularly relevant, as elevated PTH has been independently associated with sarcopenia and impaired muscle metabolism [45]. PTH signaling, through the PTH/PTHrP receptor, has been shown to stimulate muscle wasting by activating the ubiquitin–proteasome pathway [45,46].

Our findings revealed a strong positive correlation between PTH and CRP (r = 0.62), highlighting the relationship between hormonal stress and systemic inflammation. Chronic PTH elevation may promote a pro-inflammatory micro-environment [47], which supports our GLM analysis identifying CRP as a primary independent predictor of muscle mass loss. Systemic inflammation, as reflected by CRP, acts as a catabolic stimulus that inhibits protein synthesis and accelerates muscle fiber degradation [47]. This observed association indicated that elevated PTH levels may directly target skeletal muscle via PTH/PTHrP receptors, potentially stimulating the production of atrogenes such as Muscle Ring Finger 1 (MuRF1) and Muscle Atrophy F-box (MAFbx). Such processes activate the ubiquitin–proteasome pathway (UPS), leading to the breakdown of myofibrillar proteins [45,46]. Furthermore, the interaction between PTH and inflammatory markers like CRP may foster a pro-catabolic environment, where PTH-induced cytokines suppress mTOR signaling, resulting in persistent muscle wasting [47]. By reducing both PTH and CRP levels, the intervention appears to have mitigated this hormonal-inflammatory cascade, thereby preserving muscle fiber integrity.

In addition, the greater reduction in IG plasma 8-isoprostanes confirms that mediating lipid peroxidation is crucial for recovery due to oxidative stress upregulation of mitochondrial dysfunction in skeletal muscle, contributing to energy and protein loss [48]. The significant decrease in lipid peroxidation markers was consistent with the perspective of previous studies [3,29,32], which identify oxidative stress as a primary driver of myofibrillar protein breakdown. Our results also demonstrated that mitigating this oxidative impact was not only a biochemical shift but a functional necessity for maintaining muscle mass and strength in the early rehabilitation phase [48,49].

The supplement’s omega 6/omega 3 ratio of 3.1/3 ensured the low omega 6 participation on the eicosanoid inflammatory compound formulation and supported an optimal microenvironment against inflammation and peroxidation process [50]. By suppressing this inflammatory-oxidative cascade, the nutritional intervention provided a metabolic protection, playing a critical role in muscle mass retention.

On the other hand, we should mention the soy lecithin’s possible role not only as an emulsifier but as a molecular switch in the absorption of soluble micronutrients and elements. Lecithin, even in very small concentrations, acts as a source of soluble organic phosphate and enhances the solubilization of lipophilic compounds which are essential for the optimal absorption of bone-supporting micronutrients such as calcium, enhancing their bioavailability [51].

Consequently, the gains we observed were not just quantitative, but represent a qualitative reorientation our approach to geriatric orthopedic recovery. By optimizing the protein, electrolyte, and mineral environment, functional independence was improved, as seen in the increased gait speed and balance of our patients. These findings suggest that specialized nutrition could play a crucial role in geriatric care, helping older adults regain their quality of life faster and alleviating the long-term impact of postoperative disability.

The primary strength of this study was the multi-dimensional approach to musculoskeletal health, combining high-precision biochemical markers with validated functional assessments. The assessment of plasma isoprostanes and CRP levels provides a strong mechanistic link, while the application of GLM ensures statistical significance.

Regarding the psychological assessment, although depression scores were not a primary endpoint of this study, it is important to note that all participants were closely monitored by their attending physicians during the follow-up period. None of the included patients presented with severe mental or psychological disorders that could have compromised their ability to comply with the nutritional protocol or affected the recovery process. This clinical oversight ensured that the participants’ mental health status remained stable, preventing it from acting as a confounding factor in the evaluation of the intervention’s clinical outcomes.

However, certain limitations should be addressed. The single-blind nature of the study and the lack of a placebo supplement are recognized. In a clinical hospital environment, we prioritized patient transparency and the ethical right to informed consent, ensuring that participants were fully aware of the nutritional support provided for their recovery. To eliminate any potential source of bias, a rigorous investigator-blinded protocol was strictly implemented. While the trial was open-label for participants, all functional outcomes were assessed by an independent researcher who was completely unaware of group allocation. Furthermore, the statistical analysis (GLM) was performed using coded data by an investigator blinded to group identities. The fact that the supplement was administered by staff members not involved in measurements or analysis further ensures the integrity and objectivity of our results, strengthening the reliability of our findings.

Moreover, the risk of type I error is increased because the multiple analyses were conducted for secondary outcomes. Therefore, these results should be considered exploratory. Our sample size was sufficient to reach statistical significance across primary and secondary outcomes, but remains relatively small for a broader generalization of the results.

Larger, multicenter trials are needed to confirm these metabolic and functional benefits across more diverse populations. Our study also included participants undergoing either knee or hip arthroplasty due to end-stage OA. While both procedures share a common rehabilitative goal and a similar postoperative inflammatory profile, the biomechanical recovery and muscle recruitment patterns between the knee and hip joints may differ. Future studies should focus on stratifying results by site to identify potential knee or hip joint responses to the intervention.

Overall, the observed gains in muscle mass and function were not only a quantitative increase but a qualitative shift supported by reduced systemic inflammation and a highly optimized mineral and electrolyte environment. 

## 5. Conclusions

The nutritional supplementation intended to support rehabilitation by a combined targeted nutritional strategy aiming at three goals, as follows: to suppress catabolism and enhance muscle anabolism, to provide calcium and phosphates in highly bioavailable forms while maintaining low sodium and potassium levels, and to control oxidative stress and inflammation by supporting anti-inflammatory responses.

Our findings suggest that the provided nutritional strategy acted as a multifaceted biochemical shield, preserving muscle mass and integrity and restraining oxidative stress and inflammation during the critical postoperative window. By bridging the gap between molecular markers, like F2-isoprostanes and CRP, and real-world functional gains, we have shown that when we support the “muscle–bone–joint axis” nutritionally, patients do not just recover but also regain their independence faster and with less symptomatic burden.

For the clinicians, these results suggest that incorporating specialized nutritional support into conventional orthopedic protocols is an efficacious approach to enhance a standard recovery and a quality of life of an older adult population that refuses to set aside everyday living due to lower limb osteoarthritis.

## Figures and Tables

**Table 1:** Baseline characteristics of the study population (N = 88).

Characteristics	Intervention Group (n = 44)	Control Group (n = 44)	*p*-Value
Total Knee arthroplasty	26 (59.1%)	29 (65.9%)	
Tital Hip Arthroplasty	18 (40.9%)	15 (34.1%)	0.419
Age (years)	71.4 ± 6.2	72.1 ± 5.8	0.584
Gender (Male/Female)	20/24	19/25	0.945
Weight (kg)	78.5 ± 12.4	77.2 ± 11.9	0.621
BMI (kg/m^2^)	27.8 ± 4.1	27.5 ± 3.8	0.725
Comorbidities n (%)			
Diabetes	5 (11.1%)	5 (11.9%)	0.908
Dyslipidemia	16 (35.6%)	15 (35.7%)	0.988
Previous Surgery	13 (28.9%)	10 (23.8%)	0.592
Smoking	8 (17.8%)	9 (21.4%)	0.669
Depression	3 (6.7%)	11 (26.2%)	0.013 *

BMI: Body Mass Index. * Indicates statistical significance at *p* < 0.05.

**Table 2:** Mean daily energy and macronutrient intake, and proportion of protein sources throughout the 15-week intervention period.

Nutrient Intake/Day (15-Week Average)	Intervention Group (IG) (n = 44)	Control Group (CG) (n = 44)	*p*-Value
Total Energy Intake (kcal/day)	1923.63 ± 471.29	1932.72 ± 384.58	944
Total Carbohydrates (g/day)	240.45 ± 58.91	241.59 ± 48.07	943
Total Fat (g/day)	64.12 ± 15.70	64.42 ± 12.81	945
Total Protein Intake (g/day)	96.18 ± 23.56	96.63 ± 19.22	945
Total Protein Intake (g/kg/day)	1.31 ± 0.22	1.27 ± 0.17	498
—Protein from Supplement (g/day)	27.50	0.00	—
—Protein from Whole Foods (g/day)	68.68 ± 23.56	96.63 ± 19.22	<0.001
Proportion of Protein Intake (%)			
—% from Supplement	28.6%	0.0%	—
—% from Whole Food Sources	71.4%	100.0%	<0.001

**Table 3:** Adjusted effects of the intervention on body composition and biochemical markers.

Variable	Group	Baseline (Mean ± SD)	15th Week (Mean ± SD)	Adj. Mean Change (Δ)	CI 95%	*p*-Value
Primary Outcomes						
ASMM (kg)	IG	24.23 ± 6.80	26.57 ± 6.91	2.34	(1.99, 2.69)	<0.001
	CG	21.75 ± 7.78	21.52 ± 7.54	Ref.		-
ASMMI (kg/m^2^)	IG	9.03 ± 1.81	9.85 ± 1.75	0.82	(0.64, 1.00)	<0.001
	CG	8.42 ± 2.31	8.35 ± 2.25	Ref.		-
Secondary Outcomes						
Handgrip Strength (kg)	IG	23.80 ± 13.39	28.20 ± 12.11	4.40	(3.13, 5.67)	<0.001
	CG	20.15 ± 10.92	19.82 ± 11.15	Ref.		-
Gait Speed (m/s)	IG	0.82 ± 0.29	1.05 ± 0.24	0.23	(0.15, 0.31)	<0.001
	CG	0.80 ± 0.33	0.81 ± 0.31	Ref.		-
2-Minute Walk Test (m)	IG	140.13 ± 23.65	165.40 ± 22.10	12.02	(5.85, 18.2)	0.026
	CG	129.07 ± 33.59	130.15 ± 32.40	Ref.		-
5-Repetitions Sit to Stand Test (s)	IG	18.05 ± 9.70	14.12 ± 8.45	−3.93	(−5.62, 2.20)	<0.001
	CG	15.58 ± 10.50	15.75 ± 10.20	Ref.		-
SLS—Right Foot (s)	IG	11.32 ± 10.56	16.45 ± 11.20	5.13	(3.00, 7.30)	<0.001
	CG	10.97 ± 10.58	11.12 ± 10.45	Ref.		-
SLS—Left Foot (s)	IG	10.00 ± 9.00	14.85 ± 9.50	4.85	(2.99, 6.71)	<0.001
	CG	8.49 ± 7.49	8.55 ± 7.30	Ref.		-
Phase Angle (°)	IG	5.47 ± 1.90	6.12 ± 1.85	0.65	(0.41, 0.89)	<0.001
	CG	5.20 ± 2.02	5.18 ± 2.05	Ref.		-
Biochemical markers						
Plasma 8-Isoprostanes (ng/mL) (pg/mL)	IG	171.49 ± 104.81	45.55 ± 28.62	−29.19	(−39.81, −18.57)	<0.001
	CG	170.15 ± 115.40	88.73 ± 53.32	Ref.		-
CRP (mg/L)	IG	2.54 ± 5.33	1.85 ± 4.10	−0.69	(−0.98, −0.40)	0.004
	CG	2.32 ± 6.74	2.45 ± 6.50	Ref.		-
PTH (pg/mL)	IG	96.82 ± 32.15	69.41± 24.10	−27.41	(−44.46, −10.36)	<0.001
	CG	95.12 ± 28.45	94.85± 27.90	Ref		-

Models adjusted for baseline values, age, weight, and comorbidities using Generalized Linear Models (GLM); (β) = beta: represents the adjusted mean difference between groups at 15 weeks, Adj. mean change (Δ): represents the adjusted mean difference between groups at 15 weeks; IG: Intervention Group, CG: Control Group, ASMM: Appendicular Muscle Mass.; ASMMI: ASMM: Appendicular Muscle Mass Index; Blood Isoprosanes: Plasma 8-Isoprostanes; CRP: C-Reactive Protein; SLS: Single-Leg Stance. (°): refers to Phase Angle unit.

## Data Availability

The data presented in this study are not publicly available due to the sensitive nature of the participants’ clinical information and because of the strict ethical restrictions regarding privacy of patients. Furthermore, the datasets are part of an ongoing research project with additional planned analysis. Anonymized data can be submitted to the corresponding authors once the research data have been completely analyzed.

## Abbreviations

The following abbreviations are used in this manuscript:

ASMM	Appendicular Skeletal Muscle Mass
CG	Control Group
IG	Intervention Group
mTOR	Mechanistic Target of Rapamysine
ASMMI	Appendicular Skeletal Muscle Mass Index
MPC	Milk Protein Concentrate
